# DRL-GAN: A Hybrid Approach for Binary and Multiclass Network Intrusion Detection

**DOI:** 10.3390/s24092746

**Published:** 2024-04-25

**Authors:** Caroline Strickland, Muhammad Zakar, Chandrika Saha, Sareh Soltani Nejad, Noshin Tasnim, Daniel J. Lizotte, Anwar Haque

**Affiliations:** Department of Computer Science, The University of Western Ontario, London, ON N6A 3K7, Canada; mzakar@uwo.ca (M.Z.);

**Keywords:** network security, Network Intrusion Detection System, Deep Reinforcement Learning, Generative Adversarial Networks, NSL-KDD, Machine Learning

## Abstract

Our increasingly connected world continues to face an ever-growing number of network-based attacks. An Intrusion Detection System (IDS) is an essential security technology used for detecting these attacks. Although numerous Machine Learning-based IDSs have been proposed for the detection of malicious network traffic, the majority have difficulty properly detecting and classifying the more uncommon attack types. In this paper, we implement a novel hybrid technique using synthetic data produced by a Generative Adversarial Network (GAN) to use as input for training a Deep Reinforcement Learning (DRL) model. Our GAN model is trained on the NSL-KDD dataset, a publicly available collection of labeled network traffic data specifically designed to support the evaluation and benchmarking of IDSs. Ultimately, our findings demonstrate that training the DRL model on synthetic datasets generated by specific GAN models can result in better performance in correctly classifying minority classes over training on the true imbalanced dataset.

## 1. Introduction

The increasing volume and sophistication of network-based attacks motivate the development of effective techniques and tools to prevent service disruption, unauthorized access, and the disclosure of sensitive information [1]. An Intrusion Detection System (IDS) is an important defence tool against sophisticated and increasing network attacks, but these systems, especially Machine Learning (ML)-based systems, require large, reliable, and valid network traffic datasets to be effective. Many available datasets span various network attacks, traffic patterns, and include attacker infrastructure details. Unfortunately, the ever-growing diversity of modern networks means that these datasets frequently fall short in providing sufficient information to create effective classification mechanisms. These datasets often suffer from a lack of traffic diversity and volume, or fail to cover the full scope of known attack types. To cope with these changes, we require a more dynamic dataset that will improve the ability of an IDS to detect network intrusions. Using Deep Learning (DL) techniques such as a Generative Adversarial Network (GAN), we can fabricate additional data using existing datasets to increase the classification accuracy of an IDS, especially for rare attack categories.

Two methods of IDSs are the Signature-based Intrusion Detection System (SNIDS) and the Anomaly-based Intrusion Detection System (ANIDS). The SNIDS approach is effective for known threats, as it looks for specific patterns (or ‘signatures’) such as byte sequences in network traffic, or known malicious instructions sequences used by malware [1]. Conversely, the ANIDS approach uses ML algorithms to analyze and monitor the network traffic in order to detect any suspicious activity, thus being an effective method for catching unknown attacks [2].

The emergence of DL and its integration with Reinforcement Learning (RL) has created a class of Deep Reinforcement Learning (DRL) methods that are able to detect the most recent and sophisticated types of network attacks. DRL combines artificial neural networks with a framework of RL that helps software agents (or ‘learning entities’) learn how to reach their goals. DRL combines function approximation and target optimization, mapping states and actions to the rewards they lead to [3]. This results in a ‘policy’ that our learning agents can follow to make the best decisions given the current state. To detect network attacks, DRL is used to train an agent such that, given a ‘state’ represented as a collection of feature values, will take the best ‘action’ (which, in our case, acts as a classification of network traffic), in order to recognize an attack. DRL is a powerful and effective tool for network traffic classification due to its ability to extract and learn from complex features in high-dimensional data, providing a more nuanced understanding of network behavior without extensive manual feature engineering [4].

Each network is different in that its behaviours and patterns evolve gradually. Naturally, vulnerabilities also evolve. The performance of IDS classification accuracy suffers as existing datasets gradually become out of date, invalid, and unreliable. Moreover, reliable data often cannot be shared due to privacy concerns. Existing publicly available datasets do not include all of the existing network attack types, let alone the unknown vulnerabilities and attacks. To resolve this, we need more diverse and up-to-date datasets that properly reflect the characteristics of network intrusions in order to increase the performance of the IDS. Knowing this, we propose an SNIDS using DRL techniques. We use a collection of GAN models to generate varied datasets, then use DRL to implement an IDS and train the model on the GAN-generated datasets and compare our results.

Our work uses the open-source dataset NSL-KDD [5]. NSL-KDD is imbalanced with significantly less attack samples than normal traffic (especially for Probe, U2R, and R2L attacks). Thus, we use a GAN to generate synthetic data so that there is a more even class balance. We then trained a DRL model on both the untouched NSL-KDD dataset as well as the GAN-generated data from each of our unique models for both binary and multiclass classification. Finally, we assess how training the DRL models using synthetic datasets compares in terms of IDS performance as well as individual class F1-scores.

Overall, the primary contributions of this paper include:

1. Using both conditional and unconditional CTGAN and CopulaGAN models to generate tabular data. This is useful for increasing the minority class samples in imbalanced datasets, as well as providing large datasets for training ML models.
2. Combining GAN and DRL techniques for the purpose of network intrusion detection and increasing the precision and recall for classifying underrepresented class data. We propose a framework that trains a GAN model to produce synthetic data, and then uses that data to train a DRL model that acts as an IDS and either alerts the user to a specific type of attack or classifies the network traffic as benign.

The remainder of this paper is organized as follows: Section 2 surveys related work for the purpose of network intrusion detection and presents the motivation and novelty behind this work. Section 3 discusses the methodology and details necessary for implementation of our models. Section 4 provides a comprehensive evaluation of the obtained results. Section 5 presents an interpretation of our findings, limitations of our work, as well as directions for future work.

## 2. Related Work

### 2.1. DL-Based IDS Research

The first IDS was proposed by Anderson in 1980 [6]. Since then, many mature IDS solutions have been implemented. Despite advancements, many IDSs continue to deal with persistent challenges, particularly in terms of high false alarm rates and the inability to detect unknown attacks. The prevalence of false alarms not only burdens security analysts, but also increases the risk of overlooking genuinely harmful attacks. With the rapid evolution of network environments and the emergence of new attack variants, there is an urgent need for IDSs capable of identifying unknown threats. In response to these challenges, researchers have shifted away from traditional ML techniques such as Naive Bayes, Decision Trees, and Support Vector Machines, and are now exploring DL as a potential solution. DL offers the potential to automatically learn intricate patterns from raw network data, enabling IDSs to achieve higher detection rates, reduce false alarm rates, and effectively identify both known and unknown attacks in dynamic network environments [7].

DL techniques commonly employed for IDSs include convolutional neural networks for spatial pattern recognition in network traffic [8], recurrent neural networks such as LSTMs for analyzing sequential data such as system logs [9], and autoencoders for anomaly detection by learning compressed representations of normal behavior [10]. While these techniques offer solutions for detecting various cyberthreats and anomalies in diverse network environments, they often require fixed training datasets and may lack the ability to adapt dynamically to new threats.

Recognizing these limitations, recent research has leveraged DRL to dynamically adapt to evolving threats and enhance detection performance. Hsu and Matsuoka [1] proposed a DRL model for an ANIDS, where network traffic data constitutes the RL environment state variables, and the outcomes of intrusion detection form the action space. The model employs a unique approach by dynamically alternating between ‘detection mode’ and ‘learning mode’. In learning mode, detection performance is evaluated through a reward system, allowing the model to update itself with new data to improve accuracy. Detection mode uses a dummy reward to maintain operational status without calculating the true reward. This model is tested on the NSL-KDD and UNSW-NB15 [11] benchmark datasets, and achieves impressive metrics with over 90% accuracy, recall, and precision.

Building on similar principles, Alavizadeh et al. [12] and Benaddi et al. [13] developed a DRL-based IDS model tailored to different network environments. The authors introduced a continuously updating, self-learning IDS that integrates Deep Q-Learning (DQL) with a deep feedforward neural network. This system uses a trial-and-error auto-learning approach to improve detection capabilities across various network intrusions, achieving a classification accuracy of 78% on the NSL-KDD dataset, outperforming several other ML techniques. Meanwhile, Benaddi et al. presented a DRL-based IDS designed for wireless sensor networks and the Internet of Things. Given the increasing adoption of these technologies in sectors such as healthcare, business, and smart cities, they face significant challenges from cyberthreats due to inherent security flaws, zero-day vulnerabilities, and widespread accessibility. The DRL-IDS model not only enhances detection performance by monitoring real-time network traffic but also surpasses standard RL and K-Nearest Neighbours approaches in accuracy and detection rates, with fewer false negatives when assessed using the NSL-KDD dataset. These studies highlights the potential of adaptive learning capabilities in fortifying network security against sophisticated cyberattacks.

Expanding on these advancements, a novel application of several DRL algorithms to IDS was detailed by Lopez-Martin et al. [4], where a conceptual modification replaces the classic DRL paradigm of interacting with a live environment with a sampling function from recorded training intrusions. This pseudo-environment generates rewards based on detection errors during training, enabling the adaptation of algorithms such as deep Q-networks, double deep Q-networks, and actor-critic to intrusion detection tasks. Notably, the double deep Q-networks algorithm exhibit superior performance, significantly enhancing detection rates and processing speed compared to traditional models. Evaluated using the NSL-KDD and AWID [14] datasets, this approach not only proves more effective in reducing false alarms but also in detecting unknown threats, thereby marking a significant advancement in the capabilities of IDS to adapt to and counteract the rapidly evolving landscape of cyberthreats.

### 2.2. Synthetic Network Traffic Data Generation Utilizing GAN Models

In recent years, GANs have been applied to generate realistic network traffic data for various purposes, including testing the robustness of IDSs and training anomaly detection models. By learning the underlying distribution of large amounts of legitimate network traffic, GANs can produce synthetic data that closely resembles real-world traffic, enabling researchers to evaluate the effectiveness of security measures and enhance network security defenses. Additionally, GAN-generated traffic can be used to simulate diverse attack scenarios, aiding in the development and validation of intrusion detection algorithms and security solutions [15].

The use of GANs for the generation of synthetic network traffic data began to gain prominence around the mid to late 2010s. One of the early notable works in this area is by Lin et al. [16], who proposed a IDSGAN, a framework that uses GANs to generate adversarial malicious network traffic to deceive IDSs. The primary goal is to leverage GANs to improve IDSs by exposing them to new, more combative and adversarial attack methods and types. This system models the black-box analogy of IDSs from the perspective of an attacker that would generally not know about the internal details of the detection system. A generator transforms known malicious traffic records into adversarial ones and a discriminator classifies the records to learn about the originally unknown detection system. The authors demonstrated the validity of their system by only modifying the nonfunctional features of the records such that the modified records would still classify as an intrusion and not junk traffic. They evaluate their system using the standard NSL-KDD dataset on multiple different detection models including Naive Bayes, Random Forest, and multilayer perceptron classifiers. IDSGAN achieves good results, with the detection rate of the DoS attack type dropping from approximately 80% with normal records to less than 1% with modified, adversarial records.

Further advancing this field, Cheng introduced PAC-GAN [17], a convolutional neural network GAN designed to create network traffic data at the IP packet layer. Similar to its predecessors, PAC-GAN focuses on the importance of generating realistic network traffic, crucial for robust cybersecurity measures. This system goes a step further by proving its ability to generate and transmit traffic flows such as ICMP Pings, DNS queries, and HTTP web requests successfully through the Internet, receiving appropriate responses from network entities. This breakthrough demonstrates the practical applications of GANs in live network environments, confirming the potential of GANs not just to test but also to enhance real-time network security frameworks. PAC-GAN’s success lays the groundwork for future developments in GAN-based traffic generation, potentially transforming how network security is approached.

Tertsegha et al. [18] expanded the scope of synthetic network data generation by introducing three innovative flow-based network traffic generators that also leverage the Improved Wasserstein GAN (WGAN-GP) with a two-time scale update rule. These generators are adept at producing synthetic network traffic that captures the intricate internal dependencies inherent in flow-based data. The authors develop three specific methods for data synthesis: N-WGAN-GP treats IP addresses and ports as continuous values, B-WGAN-GP transforms attributes into binary formats to preserve detailed categorical information, and E-WGAN-GP employs IP2Vec to create meaningful continuous representations of categorical attributes. Their evaluation on the CIDDS-001 dataset [19] indicates that E-WGAN-GP and B-WGAN-GP excel in generating highly realistic data, while N-WGAN-GP underperforms, suggesting that simpler numeric transformations may not sufficiently capture complex network behaviors.

### 2.3. ML-Based IDSs Utilizing GAN Methods

Despite achieving high detection accuracy with ML-based and DL-based IDS techniques, many IDSs still struggle with effective training due to imbalanced datasets. The challenge is exacerbated by the relatively small size of abnormal or attack data compared to normal data in most existing datasets, leading to a significant imbalance during training. Additionally, the distribution of the data space may not be fully discernible to the IDS because of missing data, even when the data volume is adequate. To address these issues, GANs have been employed to create new synthetic samples that aim to closely resemble the original data.

Shahriar et al. [20] implemented a GAN-IDS framework model for the NSL-KDD dataset, aimed at improving performance in cyber–physical systems with limited data availability. Recognizing the potent capabilities of GANs within the DL realm and the critical role of IDS in CPS security, this integration, referred to as G-IDS, specifically addresses the challenges posed by imbalanced datasets. The model presented in this work demonstrates superior accuracy in predicting threats compared to traditional standalone IDS systems, even with minimal initial data. However, the centralized nature of G-IDS, along with its computational and time-intensive requirements, require further exploration.

Liu et al. [20] addressed the issues of imbalance and high dimensionality in datasets used for intrusion detection by proposing an oversampling technique based on GAN and feature selection. The authors used GANs to oversample underrepresented attack classes, creating a rebalanced, low-dimensional dataset suitable for training ML models, which enhance the efficiency of intrusion detection. This method was applied to the NSL-KDD, UNSW-NB15 [11], and CICIDS-2017 [21] datasets, resulting in notable improvements in the detection capabilities of ML models.

Qui et al. [22] introduced DIGFuPAS, a framework leveraging GANs to enhance the robustness of ML-based IDSs in smart city applications. This framework generates mutated cyberattack data flows that can bypass ML-IDSs, providing a method for continuously testing and improving the IDS within software defined networking environments. DIGFuPAS is evaluated on its ability to improve detection rates and F1-scores, using the CICIDS-2017 dataset. This framework serves as an automated sustainability test pipeline, facilitating ongoing assessment and enhancement of IDSs capabilities against sophisticated cyberattacks.

Kumar and Sinha [23] developed a model for generating synthetic IDS datasets using a Wasserstein Conditional GAN (WCGAN) coupled with an XGBoost classifier, incorporating a feature reduction technique via a deep autoencoder. The authors tested this model on the NSL-KDD, UNSW-NB15, and BoT-IoT datasets, achieving F1-scores of 0.96, 0.81, and 0.99, respectively. However, the evaluation of the effectiveness of WCGAN on ML classifiers involve a combination of real and synthetic samples rather than solely using synthetic data for training. The features are also inconsistent across the datasets, and the observed high detection performance was primarily restricted to the NSL-KDD dataset.

While the aforementioned studies primarily address enhancements in IDS through dataset quality and static model accuracy improvements, our work introduces a comprehensive approach by integrating both conditional and unconditional CopulaGAN and CTGAN models with a DRL-based IDS. This combination targets the pressing issues of insufficient and imbalanced datasets. Our model leverages the advanced capabilities of DRL to refine the response strategies of the system, enhancing its ability to detect minority attack types more accurately than if trained on the original unbalanced dataset. By using synthetic data generated by CopulaGAN and CTGAN models into the process of training our DRL agent, our approach markedly advances beyond conventional methods. This strategy provides a solution that improves the ability of the system to classify underrepresented classes and react to diverse cyberthreats effectively.

## 3. Methods

### 3.1. NSL-KDD Dataset

NSL-KDD is an updated version of the KDD’99 dataset [5]. Basic processing has been completed, such as the removal of redundant records preventing classifiers from becoming biased towards more frequent records. The use of the NSL-KDD dataset has been very popular in studies on IDSs, in a sense, becoming the de facto standard. It contains information which can help to build a host-based and network-based intrusion detection model to ensure network security in a variety of systems.

**Figure 1:**
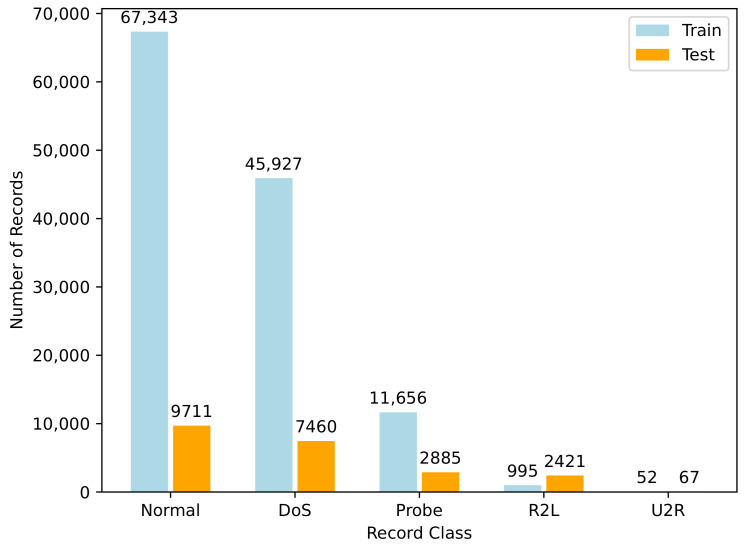
Distribution of NSL-KDD dataset by record classes.

The training and test set contains 125,973 and 22,544 records, respectively. This includes 42 features; however, we remove ‘Num outbound cmds’ as all records contain 0, so we are left with 41 features: 9 basic features, 12 content features for the connection, 9 temporal features calculated at two-second time windows, 10 statistical network traffic features, and 1 class label. Table 1 lists the features present in the dataset. The training set contains 22 attack types and the test set contains 37 attacks types. The 15 attack types not included in the training set make this dataset excellent for modeling unknown attacks. We opt to use the common five-class classification of network traffic records: normal, DoS, Probe, R2L, and U2R. Table 2 describes these five classes in further detail (with the class ID referring to the numerical mapping used by both the DRL and GAN models). The distribution of record classes can also be seen in Figure 1.

### 3.2. Machine Learning Performance Evaluation

We used the accuracy and F1-score (which combines precision and recall) metrics to evaluate the performance our DRL model and other ML algorithms. While the accuracy score only measures the percentage of correctly classified samples, this selection of performance metrics allows us to evaluate the percentage of samples that were incorrectly classified. This is especially important for IDSs as the accuracy performance metric is not enough to evaluate imbalanced datasets, such as network traffic data, which generally include significantly more normal traffic. These performance metrics are derived from the True Positive (TP), True Negative (TN), False Positive (FP), and False Negative (FN) values.

#### 3.2.1. Accuracy

Accuracy measures the number of correct predictions out of the total predictions made by the model. In this case, accuracy measures the model’s ability to correctly identify normal and attack traffic records. Equation (1) formalizes the accuracy performance metric:

(1) $$\mathrm{Accuracy}=\frac{\mathrm{TP}+\mathrm{TN}}{\mathrm{TP}+\mathrm{FP}+\mathrm{TN}+\mathrm{FN}}$$

#### 3.2.2. Precision

Precision measures the number of correct positive predictions out of the total number of positive predictions. In this case, precision measures the model’s degree of correctness in predicting attack records over the total number of attacks predicted [1,24,25]. Equation (2) formalizes the precision performance metric:

(2) $$\mathrm{Precision}=\frac{\mathrm{TP}}{\mathrm{TP}+\mathrm{FP}}$$

#### 3.2.3. Recall

Recall measures the number of correct positive predictions out of the total number of positive instances in the dataset. In this case, recall measures the model’s ability to correctly identify attack traffic records. From this definition, recall is also referred to as the true positive rate, detection rate, or sensitivity. Equation (3) formalizes the recall performance metric:

(3) $$\mathrm{Recall}=\frac{\mathrm{TP}}{\mathrm{TP}+\mathrm{FN}}$$

#### 3.2.4. F1-Score

F1-score is the harmonic mean of the precision and recall values, essentially a combined measure of the two performance metrics. F1-score quantifies how discriminative the model is [26] and acts as a good indicator of performance since a decrease in either precision or recall results in a significant decrease in the F1-score. In addition, for multiclass classification, we present both the unweighted and weighted F1-scores. The weighted F1-score accounts for label imbalance by considering the number of instances of each label when calculating the average F1-score. Equation (4) shows how the F1-score is calculated:

(4) $$\begin{array}{cc} F1\;\mathrm{Score} & =2\cdot \frac{Precision\cdot Recall}{Precision+Recall}\\ \; & =\frac{TP}{TP+\frac{1}{2}\left(FP+FN\right)} \end{array}$$

### 3.3. Statistical Evaluation of Synthetic Data

To evaluate the synthetic data generated by the GAN models against the real data they were trained on, we used statistical metrics that compare the columns of the synthetic tables against those in the real tables. These statistical metrics are as follows.

#### 3.3.1. CSTest

The CSTest compares columns with discrete values using the Chi-squared test to compare their distributions. The output of the test is an average of the CSTest *p*-values for each of the columns, which ultimately quantifies the probability that the compared columns were sampled from the same distribution.

#### 3.3.2. KSTest

The KSTest compares columns with continuous values using the two-sample Kolmogorov–Smirnov test and empirical Cumulative Distributed Function (CDF) to compare their distributions. The output of the test is an average of 1 minus the KSTest D statistic for each of the columns. This result quantifies the maximum distance between the CDF expected and observed values.

#### 3.3.3. KSTestExtended

The KSTestExtended is an extension of the KSTest that converts all columns to numerical values using a hypertransformer and then applies the regular KSTest.

### 3.4. Detection-Based Evaluation of Synthetic Data

Detection metrics use ML models to determine how distinguishable the synthetic data are from the real data. To achieve this, both the synthetic and real tables are shuffled and a flag indicating whether the record is synthetic or not is added. Next, cross-validation is used with a selected ML model that predicts the flag, outputting 1 minus the average ROC AUC of all the cross-validation splits. Because the ROC AUC measures the separability of the classes from the model, a high detection metric score means that the model is unable to easily distinguish the synthetic records from the real ones.

### 3.5. Generative Adversarial Network Models

**Figure 2:**
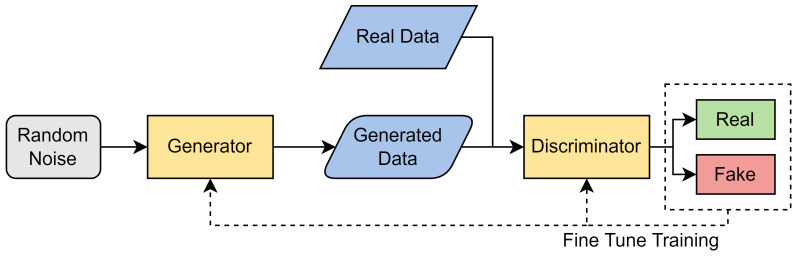
Architecture of GANs.

Goodfellow et al. [27] first proposed the idea of a GAN in 2014 as an unsupervised learning method that generates synthetic data using an input of real data. GANs are used to generate realistic synthetic data using real data, usually because obtaining more data can be difficult, time consuming, and costly. GANs use two independent models, a generator and a discriminator. By detecting patterns or similarity from given input data, the generator processes input data and produces more data. The discriminator is a classifier which determines the difference between the real data and the generated data. It produces a probability between 0 and 1 to define whether an instance belongs to the real data (closer to 1) or to the generated data (closer to 0). Figure 2 highlights the general workflow of GANs.

This work concentrates on CTGAN [28] and CopulaGAN, which are specialized GAN models designed for generating synthetic tabular data. These GAN models closely mimic the statistical properties of real datasets; however, they cater to slightly different needs and technical challenges, which we describe below.

Our work generates these models using implementations provided by the SDV open-source library [29], following work by Bourou et al. [30], which showed promising results for generating network traffic data using models from this library. These models were trained on the NSL-KDD training data for 100 epochs with a batch size of 500. Both models used discriminator steps of 5, matching WGAN [31], an extended version of vanilla GAN. The full list of hyperparameters and their values are given in Table 3.

The trained CTGAN and CopulaGAN models were each used to generate two synthetic datasets:

1. A dataset containing 200,000 records generated through regular sampling without conditions.
2. A dataset containing 20,000 records for each class generated using conditional sampling through rejection. These datasets were used to explore the efficacy of using GANs to generate a balanced distribution from an highly imbalanced training distribution.

#### 3.5.1. CTGAN

CTGAN introduces significant advancements to address the specific challenges of applying GANs to tabular data. Notably, it handles non-Gaussian and multimodal distributions through a mode-specific normalization technique. This approach transforms continuous values from any distribution into a bounded vector, making them more suitable for neural network processing. In contrast, earlier models normalized continuous values to the range of [−1,1] using min–max normalization. CTGAN employs a variational Gaussian mixture model to process each continuous column independently.

The CTGAN model incorporates a conditional generator and a training-by-sampling strategy to address the potential issue of data imbalance. During training, data are sampled so that all categories of discrete columns are equally represented. A conditional vector, created through one-hot encoding, specifies the value of a particular column. This conditional vector, along with random noise, serves as input to the conditional generator, *G*, designed to produce outputs that replicate the specified conditions. The training process utilizes WGAN loss with a gradient penalty to optimize the generator. The effectiveness of the conditional generator is assessed by a critic that measures the discrepancy between the model’s learned conditional distribution and the actual data distribution [30].

#### 3.5.2. CopulaGAN

The CopulaGAN model, a derivative of CTGAN, utilizes a CDF-based transformation. This modification facilitates easier learning of data by leveraging copulas [32] to model the interdependence among random variables. During training, CopulaGAN processes various data types and formats, converting nonnumerical and null data into a fully numerical format through a reversible data transformation. This enables the model to learn the probability distributions for each table column effectively. CopulaGAN also focuses on understanding the correlations between different table columns [30].

### 3.6. Deep Reinforcement Learning Model

DRL is a subfield of ML that merges RL with DL techniques. RL focuses on learning optimal policies through interaction with an environment, aiming to maximize cumulative rewards. DRL enhances this by using DL to process unstructured input data, allowing agents to make decisions without manually crafted state spaces.

In RL, problems with small, discrete state–action spaces can manage state–action mappings using a simple table, which approximates the mappings with a reasonable degree of error. However, when faced with large state–action spaces, traditional RL struggles with memory and performance due to the impracticality of storing extensive data. DRL addresses these challenges by integrating deep neural networks, which approximate value or policy functions more efficiently. Unlike traditional tables, neural networks in DRL learn to map states to values directly, enabling them to independently learn and optimize for long-term rewards in environments with large and complex state and action spaces.

The DRL models in our work were implemented using OpenAI Gym [33], stable-baselines3 [34], and Tensorflow [35]. Training of the model was done in two distinct stages to investigate the variation in performance—binary classification and multiclass classification.

#### 3.6.1. Binary Classification

For binary classification, we used an action space of size two (‘alert’ or ‘no alert’). While binary classification offers the user less knowledge on attack type specifications, it performs the basic task of an IDS—alerting the user to an attack.

**Figure 3:**
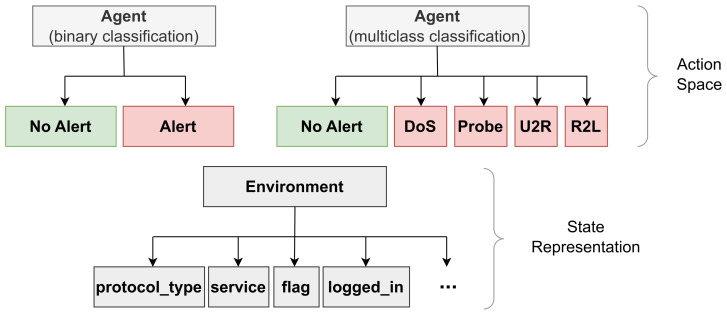
The action space and state representation of both the proposed DRL binary classification and multiclass classification model.

For binary classification, we have defined our action space, also seen in Figure 3, as follows:

- 0: No Alert (benign);
- 1: Alert (attack).

Moreover, the rewards for this model are defined by:

- +1 if agent correctly alerts to an attack;
- 0 if agent does not raise an alert when it is not needed;
−1 if agent does not raise an alert when there is an attack;
−1 if agent raises alert when there is not one needed.

In addition, we have assigned two distinct conditions for terminating an episode. An episode will be terminated if (1) it reaches a set timestep threshold or (2) if an attack is issued and no alert has been made.

The reward function for a binary IDS effectively reinforces accurate detection while penalizing both false alarms and missed attacks. By offering +1 for correct alerts and −1 for inappropriate or missed alerts, it encourages the system to maintain a critical balance between sensitivity (detecting attacks) and specificity (avoiding false alarms).

The state space is a collection of 40 features, both numerical and nominal, existing within the NSL-KDD dataset. These features can be seen in Table 1. It is important to note that features F20 and F42 are removed from the state representation, as F20 is dismissed during preprocessing, and F42 is the class label.

Initially, we trained the DRL model on the original NSL-KDD training set, described in detail above. We did this to create a baseline to determine how well our synthetic GAN-generated data performed in comparison. Prior to training our model, we converted all class labels using a binary mapping. If the class was originally ‘normal’, we assigned it a value of ‘0’; otherwise, it was assigned a value of ‘1’, implying that the data point was an attack of some sort.

For our DL models, Proximal Policy Pptimization (PPO), a policy-gradient algorithm that directly optimizes the expected reward by estimating the gradient of the policy from the trajectories taken by the agent, is employed. We applied a custom multi-layer perceptron, a class of feedforward neural network [36], of three layers with size 128, 64, and 32. In addition, each model used a rectified linear unit (ReLU) activation function. The hyperparameters used for this model can be seen in Table 4.

#### 3.6.2. Multiclass Classification

We also trained DRL models to perform multiclass classification. Similar to binary classification, we are still detecting whether there is an attack or not; however, we now attempt to classify which type of attack is taking place. Instead of ‘0’ or ‘1’, our action space consists of 0, 1, 2, 3, and 4. As stated previously, 0 maps to ‘benign’, whereas 1, 2, 3, and 4 map to DoS, Probe, R2L, and U2R, respectively. As our action space has increased in comparison to binary classification, our problem becomes significantly moire complex.

For multiclass classification, we have defined our action space, also seen in Figure 3, as follows:

- 0: No Alert (benign);
- 1: DoS;
- 2: Probe;
- 3: R2L;
- 4: U2R.

Moreover, the rewards for this model are defined by:

- +1 if agent correctly alerts to the correct type of attack;
- 0 if agent does not raise an alert when it is not needed;
−1 if agent does not raise an alert when there is an attack;
−1 if agent raises alert when there is not one needed;
−1 if agent raises an alert to the incorrect type of attack.

Both episode termination and state representation remain consistent with the description provided in Section 3.6.1.

As in binary classification, we used a ReLU activation function for multiclass classification; however, for the conditional versions of both CTGAN and CopulaGAN, we used a Sigmoid activation function, as we found that this results in a significant increase in performance on test data. For each model, we again used a custom multi-layer perceptron of three layers with size 128, 64, and 32. The hyperparameters used for this model can be seen in Table 4.

**Figure 4:**
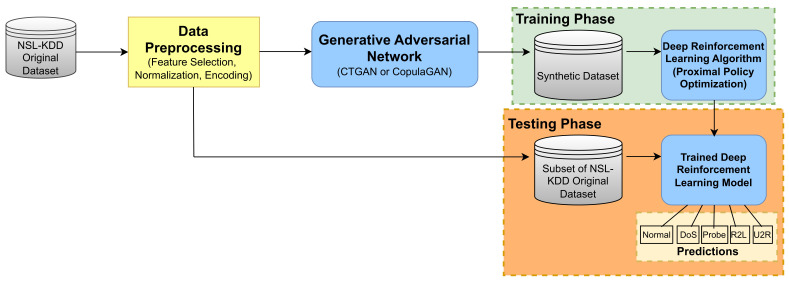
Overview of system components and interactions for multiclass classification.

An overview of the way in which each of the aforementioned components of our proposed system interact within our study can be seen in Figure 4.

## 4. Results

The following subsections describe the experimental results from our proposed GAN and DRL models, followed by a comparative analysis of our proposed model with other state-of-the-art ML methods.

### 4.1. GAN Models

The statistical metric results showcased in Table 5 indicate that both CTGAN and CopulaGAN model the discrete and continuous features of the NSL-KDD dataset effectively. As dictated by the KSTest and KSTestExtended, CopulaGAN models continuous features better than CTGAN and maintains parity for discrete features as indicated by the CSTest. Table 6 highlights the results for a linear regression classifier used to evaluate detection performance of the synthetic data. Altogether, the classifier found it challenging to distinguish the synthetic records from the real ones, which indicates that the GANs are able to capture aspects of the true dataset. Table 7 and Table 8 showcase the performance of ML models when trained to distinguish between various real and synthetic datasets. Across the board, there is comparable performance between the original real NSL-KDD dataset and the CTGAN and CopulaGAN synthetic datasets. Thus, there is promise in using synthetic data in place of real data.

### 4.2. DRL Models

#### 4.2.1. Binary Classification

**Figure 5:**
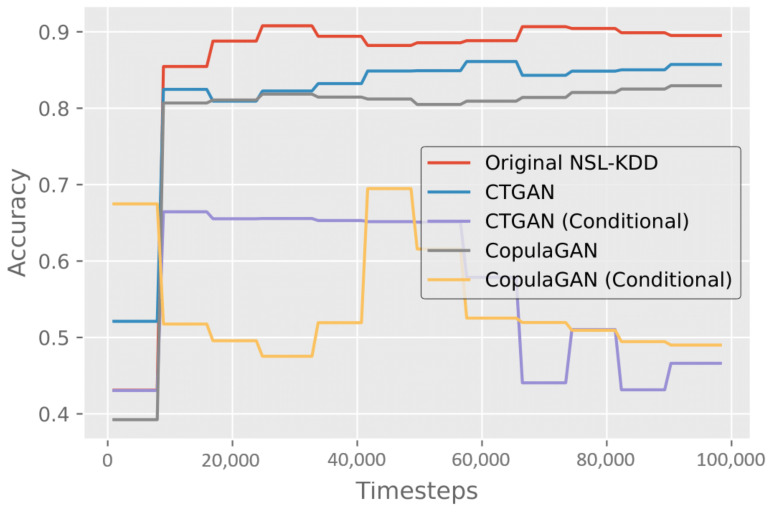
Results measuring the accuracy of binary classification after training the DRL model on both the original NSL-KDD dataset and each of the synthetic GAN datasets.

Training on the NSL-KDD training dataset for 100,000 timesteps resulted in an average accuracy of 89.5% and an F1-score of 0.906 on the test dataset, as shown in Table 7. We then trained the DRL model on each of the GAN-generated datasets one-by-one and evaluated them individually on the NSL-KDD test dataset. The detailed results of these experiments can be seen in Table 7, and viewed in terms of progressive performance for average accuracy in Figure 5.

Training on CTGAN synthetic data performs the best after the NSL-KDD trained model, with 85.7% accuracy and an F1-score of 0.869. Training using CopulaGAN synthetic data trails close behind with 82.9% accuracy and an F1-score of 0.838. The conditional variations of both CopulaGAN and CTGAN perform significantly worse than the three other datasets, reaching their peak of 70% and 66%, respectively, almost immediately, and then dropping to just below 50%.

#### 4.2.2. Multiclass Classification

Training on the NSL-KDD training dataset for 2,000,000 timesteps resulted in an accuracy of 73% and a weighted F1-score of 68.9% on the test dataset, as shown in Table 8.

We then trained the DRL model on the four GAN-generated synthetic datasets previously discussed. The most promising results were seen in training the model on CopulaGAN. The model reaches an accuracy of 70.2% and a weighted F1-score of 63%. This is just a 2.7% drop in accuracy from training on the true NSL-KDD data. Training the DRL model on the remaining three synthetic datasets underperforms when compared to both the decision tree and MLP classifier.

As discussed previously, an F1-score refers to both precision and recall being high. When we train on imbalanced datasets, the F1-scores in minority classes are typically quite low, as the ML model does a poor job of recognizing and properly classifying that test data. Looking at Table 9, we can see the F1-scores for each individual class for each of our training sets. Since NSL-KDD had extremely low records for both R2L and U2R, we can see that the F1-scores for these classes are also quite low at 0.1490 and 0.0, respectively.

One of the major goals of our work was to determine if, by generating synthetic GAN data, we could inflate the F1-scores (more specifically, precision and recall) of the minority classes from our imbalanced dataset. In Table 9, we can see that training our DRL model with data generated from conditional CTGAN and conditional CopulaGAN improved upon the F1-scores for both R2L and U2R in the same way that we would expect to see if the true dataset naturally contained more records of these two class types. Training the DRL model on synthetic data from conditional CTGAN increased the F1-scores for R2L and U2R by 0.573 and 0.172, respectively. Training on synthetic data from conditional CopulaGAN improved the F1-scores for R2L and U2R by 0.210 and 0.051, respectively. This demonstrates that the concept of using GAN models to generate synthetic data for a minority class and artificially inflating the training set in order to have better performance in classifying underrepresented classes is a viable option.

## 5. Conclusions

In this paper, we have proposed an SNIDS which is able to perform binary and multiclass classification on network traffic data using a DRL-based system. The model was trained using the publicly available NSL-KDD dataset, allowing it to detect a range of attack types on a network. To enhance the learning capabilities of our proposed model, GANs were used to fabricate training data for our DRL-based IDS. Our results demonstrate that this system is able to interact with the network and identify attack classes with competitive accuracy. In addition, we show that generating synthetic data for underrepresented classes can improve the precision and recall within these classes, thus acting as a potential solution for imbalanced datasets.

For binary classification, we obtained an 89.5% accuracy after training on the original NLS-KDD dataset. We consider this our baseline model. When trained on the four synthetic datasets, data generated from unconditional CTGAN produced an accuracy of 85.7%, the closest competition to the baseline model.

In multiclass classification tasks, our model achieved an accuracy of 73.0% when trained on the original NSL-KDD dataset. When utilizing the four synthetic datasets, CopulaGAN-generated data yielded an accuracy of 70.2%, proving to be the closest competitor to the baseline model. Therefore, our GAN-based approach successfully generates realistic data suitable for training competitive IDSs.

**Figure 6:**
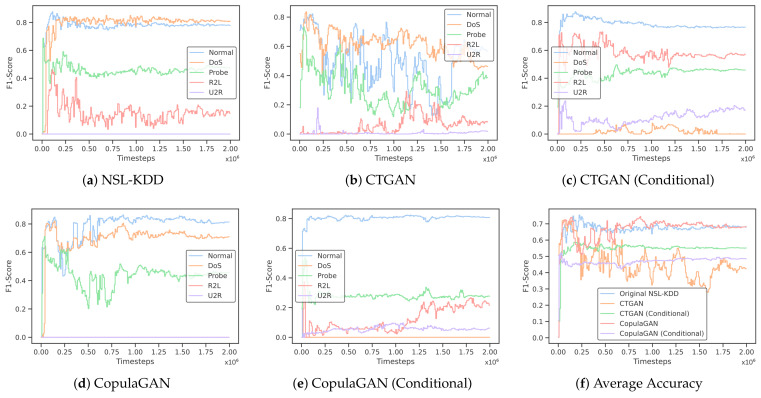
Results measuring the F1-scores of multiclass classification (**a**–**e**) as well as the averages (**f**) after training the DRL model for 2 million timesteps on both NSL-KDD as well as each synthetic dataset.

Furthermore, both Table 9 and Figure 6 demonstrate an increase in F1-scores for minority classes on the IDS trained using GAN-generated data. Thus, while our overall accuracy decreased, we are getting better precision and recall performance for the classes without sufficient data in the NSL-KDD dataset. A decrease in overall accuracy accompanied by an increase in F1-scores for underrepresented attack types is a promising outcome, particularly highlighting the enhanced capability of our model to identify less frequent, critical events. This indicates a shift towards a more balanced approach in handling class imbalances, where the improved precision and recall for minority classes are crucial. Thus, the trade-off in overall accuracy is justified by the substantial gains in effectively identifying these critical events.

Despite its widespread use, the NSL-KDD dataset presents several limitations when used for training DL models. First, the static nature of the dataset means it may not capture the dynamic and evolving patterns of new network threats, potentially leading to models that perform well on historical data, but are less effective against contemporary or future attacks. Furthermore, inherent biases and incomplete representations of attack types within the dataset can result in synthetic data that does not adequately reflect real-world complexities, thus limiting the generalizability of the trained model.

To mitigate the discussed limitations, our future work aims to expand the scope of our research by testing our proposed approach on datasets beyond NSL-KDD. We plan to explore other benchmark datasets, such as CICIDS2017, UNSW-NB15, and the DARPA Intrusion Detection Evaluation Dataset, to evaluate the robustness of our approach across diverse network environments. By testing on multiple unique datasets, we can gain insights into the adaptability of our method to different network architectures, traffic patterns, and attack scenarios, thus providing a more comprehensive understanding of its efficacy in enhancing IDSs. Additionally, conducting experiments on a variety of different datasets enables us to identify potential challenges and fine-tune our model to achieve better performance across various domains, ultimately advancing the applicability of our proposed approach in real-world cybersecurity applications.

## Figures and Tables

**Table 1:** NSL-KDD dataset features.

F#	Feature	F#	Feature
F1	Duration	F22	Is guest login
F2	Protocol_type	F23	Count
F3	Service	F24	Srv count
F4	Flag	F25	Serror rate
F5	Src bytes	F26	Srv serror rate
F6	Dst bytes	F27	Rerror rate
F7	Land	F28	Srv rerror rate
F8	Wrong fragment	F29	Same srv rate
F9	Urgent	F30	Diff srv rate
F10	Hot	F31	Srv diff host rate
F11	Num_failed_logins	F32	Dst host count
F12	Logged_in	F33	Dst host srv count
F13	Num compromised	F34	Dst host same srv rate
F14	Root shell	F35	Dst host diff srv rate
F15	Su attempted	F36	Dst host same src port rate
F16	Num root	F37	Dst host srv diff host rate
F17	Num file creations	F38	Dst host serror rate
F18	Num shells	F39	Dst host srv serror rate
F19	Num access files	F40	Dst host rerror rate
F20 *	Num outbound cmds	F41	Dst host srv rerror rate
F21	Is host login	F42	Class label

* Removed during data preprocessing.

**Table 2:** NSL-KDD dataset record classes.

ID	Class	Symbol	# of Records	Definition
0	Normal	N	77,054	Normal network traffic record
1	DoS	D	53,387	Denial of Service attack to prevent requests from intended users from being fulfilled
2	Probe	P	14,077	Probing attack to gather information such as vulnerabilities about the target machine or network
3	R2L	R	3880	An attacker tries to gain local access by sending packets to a remote machine
4	U2R	U	119	An attacker with normal access tries to gain access to the root by exploiting system vulnerabilities

**Table 3:** Hyperparameters for the trained GAN models.

Hyperparameter	Value
epochs	100
batch_size	500
embedding_dim	128
generator_dim	(256, 256)
discriminator_dim	(256, 256)
generator_lr	0.0002
generator_decay	0.000001
discriminator_lr	0.0002
discriminator_decay	0.000001
discriminator_steps	5

**Table 4:** Hyperparameters for PPO models in binary and multiclass classification.

Hyperparameter	Value
gamma	0.9
n_steps	512
ent_coef	0.00001
learning_rate	Adjusts dynamically from 0.0021 to 0
vf_coef	0.6
max_grad_norm	0.8
lam	0.8
nminibatches	16
noptepochs	55
cliprange	0.2

**Table 5:** Statistical metrics for synthetic data.

Synthetic Data	CSTest	KSTest	KSTest Extended
CTGAN	0.9971	0.9156	0.9181
CTGAN (Conditional)	0.7468	0.8655	0.8571
CopulaGAN	0.9988	0.9550	0.9574
CopulaGAN (Conditional)	0.6982	0.9000	0.8864

**Table 6:** Results for synthetic data using logistic regression.

Synthetic Data	Discernment Metric
CTGAN	0.7579
CTGAN (Conditional)	0.4139
CopulaGAN	0.6862
CopulaGAN (Conditional)	0.3948

**Table 7:** Machine Learning performance for binary classification.

Training Data	Decision Tree		AdaBoost Classifier		Logistic Regression Classifier		MLP Classifier		Proposed DRL	
	Accuracy	F1	Accuracy	F1	Accuracy	F1	Accuracy	F1	Accuracy	F1
NSL-KDD	0.8407	0.8414	0.8221	0.8270	0.8700	0.8802	0.8054	0.8080	0.8951	0.9064
CTGAN	0.8074	0.8112	0.8404	0.8486	0.8610	0.8710	0.8461	0.8545	0.8572	0.8687
CTGAN (Conditional)	0.8801	0.8927	0.9086	0.9226	0.8740	0.8853	0.9077	0.9220	0.4662	0.1172
CopulaGAN	0.7735	0.7607	0.8259	0.8246	0.8163	0.8201	0.7918	0.7831	0.8294	0.8375
CopulaGAN (Conditional)	0.8287	0.8333	0.8743	0.8881	0.8256	0.8311	0.8947	0.9074	0.4901	0.1893

**Table 8:** Machine Learning performance for multiclass classification.

Training Data	Decision Tree			MLP Classifier			Proposed DRL		
	Accuracy	F1	F1 (Weighted)	Accuracy	F1	F1 (Weighted)	Accuracy	F1	F1 (Weighted)
NSL-KDD	0.7685	0.5585	0.7338	0.7856	0.6302	0.7556	0.7300	0.4880	0.6891
CTGAN	0.7475	0.5297	0.7336	0.7765	0.6467	0.7572	0.4247	0.3033	0.4503
CTGAN (Conditional)	0.6200	0.4475	0.6525	0.7442	0.5643	0.7791	0.5520	0.3938	0.4533
CopulaGAN	0.7031	0.4165	0.6618	0.7374	0.4606	0.6863	0.7023	0.3967	0.6345
CopulaGAN (Conditional)	0.6116	0.3810	0.6215	0.7088	0.4401	0.6883	0.4839	0.2716	0.4049

**Table 9:** Class-based F1-scores for multiclass classification.

Dataset	Normal	DoS	Probe	R2L	U2R
NSL-KDD	0.7785	0.8072	0.4752	0.1490	0.0
CTGAN	0.5670	0.4618	0.3858	0.0831	0.0192
CTGAN (Conditional)	0.7662	0.0	0.4589	0.5725	0.1716
CopulaGAN	0.8139	0.7101	0.4593	0.0	0.0
CopulaGAN (Conditional)	0.8039	0.0	0.2201	0.2097	0.0512

## Data Availability

NSL-KDD dataset: https://www.unb.ca/cic/datasets/nsl.html (accessed on 19 March 2022).

## Abbreviations

**Acronym**	**Definition**
ANIDS	Anomaly Network Intrusion Detection System
CDF	Cumulative Distribution Function
DL	Deep Learning
DQL	Deep Q-Learning
DRL	Deep Reinforcement Learning
GAN	Generative Adversarial Network
IDS	Intrusion Detection System
ML	Machine Learning
PPO	Proximal Policy Pptimization
RL	Reinforcement Learning
SNIDS	Signature-based Network Intrusion Detection System
WCGAN	Wasserstein Conditional Generative Adversarial Network
WGAN-GP	Improved Wasserstein Generative Adversarial Network
